# Organometallic synthesis, reactivity and catalysis in the solid state using well-defined single-site species

**DOI:** 10.1098/rsta.2014.0187

**Published:** 2015-03-13

**Authors:** Sebastian D. Pike, Andrew S. Weller

**Affiliations:** Department of Chemistry, University of Oxford, Mansfield Road, Oxford UK1 3TA, UK

**Keywords:** solid state, organometallic, reactivity, catalysis

## Abstract

Acting as a bridge between the heterogeneous and homogeneous realms, the use of discrete, well-defined, solid-state organometallic complexes for synthesis and catalysis is a remarkably undeveloped field. Here, we present a review of this topic, focusing on describing the key transformations that can be observed at a transition-metal centre, as well as the use of *well-defined* organometallic complexes in the solid state as catalysts. There is a particular focus upon gas–solid reactivity/catalysis and single-crystal-to-single-crystal transformations.

## 1. Introduction

Organometallic chemistry, rigorously defined by the chemical synthesis and reactivity of molecules with metal–carbon bonds, is a vibrant area of research with a large variety of practical applications [1]. For example, organometallic complexes are commonly used as catalysts for the production of commodity chemicals, materials such as polymers, and in fine chemical synthesis and medicinal chemistry discovery [1,2]. The majority of discoveries in the area have been performed in the solution phase, with studies in the solid state generally often reserved only for structural analysis; for example, single-crystal X-ray crystallography and, to a significantly lesser extent, solid-state nuclear magnetic resonance spectroscopy. By contrast to the solution phase, studies on the synthesis of, and catalysis using, organometallic complexes in the solid phase have attracted significantly less attention, even though there are potential benefits to this approach, such as: improved selectivities in synthesis that comes from spatially confined environments, improved isolated yields of products and the attenuation of decomposition pathways allowing for products that might be kinetically unstable in solution to be observed in the solid state.

Heterogeneous catalysis using the surfaces of metals or ionic platform materials is a well-researched area of chemistry with many industrial uses [3–5]. However, the mechanism of the binding, catalytic steps and product release may be multifarious or difficult to resolve when using such an extended material. There may also be a limited number of ‘active sites’ for catalysis. By contrast, homogeneous catalysis tends to be significantly more well defined, allowing for the mechanistic pathways to be readily probed using a wide variety of analytical and kinetic techniques, and often uses a single active metal centre that can be tuned by modifying the supporting ligand set, often with exquisite control with regard to reactivity and selectivity [1,6]. This greater degree of molecular control using a single-site catalyst can thus allow for stereoselective or regiospecific reactivity to be more readily tuned [7,8]. Moreover, the solvent (or sometimes ligand, for example M⋯H−C agostic interactions [9]) can play a role in stabilizing the active site(s) on the metal often necessary for reactivity, by forming weak interactions that are readily replaced by incoming substrates, so-called ‘virtual’ [10] or operationally unsaturated vacant sites [11].

**Figure 1.**
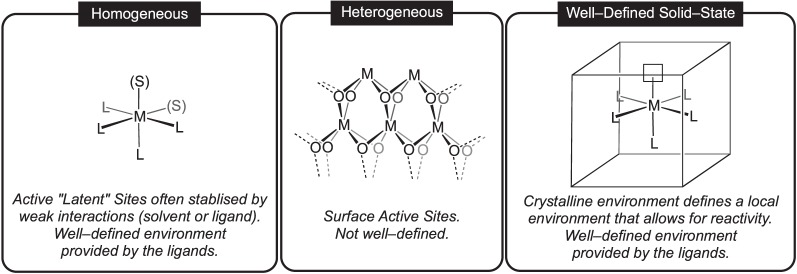
Molecular, heterogeneous and well-defined solid-state organometallic motifs.

Acting as a bridge between the heterogeneous and homogeneous realms, the use of discrete, well-defined, solid-state organometallic complexes for synthesis and catalysis is a remarkably undeveloped field (figure 1) [12–14]. In principle, if using reagents in the gas phase that can penetrate the lattice (i.e. a solid–gas reaction), or when in contact with a solvent that will not dissolve the organometallic species but solvates substrates and products, the active organometallic species can partake in the same processes observed in the solution phase, such as ligand substitution, oxidative addition, reductive elimination and insertion reactions. These resulting single-site catalysts thus bring together the benefits of heterogeneous catalysis (i.e. recyclability and easy removal from the reaction mixture) with the potential for intimate control over the transformations that the metal–ligand environment promotes in a homogeneous system [15,16]; for example, the high degrees of selectivity and mechanistic control associated with a well-defined metal–ligand environment, while harnessing the particular benefits of a solid-state environment. Further advantages of such an approach in catalysis include the simple separation of products from the catalyst [17,18], while avoiding the use of solvents in such solid–gas processes has potential economic and environmental benefits. Finally, and perhaps most excitingly, such a methodology potentially allows for the study of organometallic species without the complications of unwanted reactivity with the solvent. Such species, if being both low coordinate and of low electron count, could well be intermediates in catalytic processes that are often implicated but not observed in solution-phase chemistry. Solid-state reactivity using well-defined single-site organometallic complexes thus potentially allows for the isolation of otherwise unstable complexes, kinetically trapped in the solid state.

## 2. Scope of this review

In this review, we discuss selected examples of solid-state organometallic synthetic chemistry and catalysis, focusing on describing the key transformations that can be observed, as well as the use of *well-defined* organometallic complexes in the solid state as catalysts. In particular, we focus on gas–solid reactivity, as this presents the ideal opportunity to explore transformations and catalysis while retaining the crystalline integrity (i.e. single-crystal-to-single-crystal transformations)—which is important for both structural studies (i.e. molecular structures by single-crystal X-ray diffraction) and reactivity (well-defined voids and channels in the lattice to allow for reactivity). We do not attempt to comprehensively review the solid-state organometallic chemistry associated with simple ligand substitution reactions where crystallinity is lost [12], isomerization reactions [19] or mechanochemical transformations [12,19–23]. In addition, the vibrant field of metal–organic framework materials and the encapsulation of active species inside the cavities of these materials is only discussed in passing where appropriate [24,25]. Likewise, we do not cover photocrystallographic techniques that allow for the determination of molecular structures of metastable species generated in the crystal photochemically. This technique has been used, for example, to study linkage isomerization in transition-metal complexes with nitrosyl, dinitrogen, sulfur dioxide and nitrite ligands, when photoactivated by light of an appropriate wavelength [26]; or photoinduced spin-crossover transitions, the structural consequences of which can be measured by photocrystallography [27]. Related to this is the development of ‘crystalline molecular flasks’ [28,29] in which self-assembled cages (e.g. from Pd^2+^ ions and triazene ligands) can be used to encapsulate and stabilize highly reactive complexes formed by photo-irradiation, such as the photodissociated complex (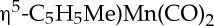 [30].

This is not the first time the general area of solid-state organometallic chemistry has been reviewed. An excellent in-depth account by Coville & Cheng [12] presented the area of solid-state organometallic chemistry in 1998 with updates with regard to isomerization reactions [19] and organometallic reactions that occur in the melt [31]. A recent (2011), short, highlight article by van der Boom [32] describes molecular single-crystal-to-single-crystal transformations in coordination chemistry. Our intention is that this review builds on these contributions, in particular bringing together both recent and older publications on solid–gas reactivity.

## 3. General considerations

For reactivity to occur in the solid state, the reagents must be able to penetrate the extended structure and access the metal sites; and this suggests that porosity within the extended solid structure will aid reaction [13,33]. The idea of a ‘reaction cavity’ was pioneered by Cohen & Schmidt [34], who proposed that reactions in the solid state occur with the least amount of molecular movement possible. There is, however, good evidence of limited molecular movement within the solid state from X-ray diffraction studies, in particular the rotation of CH_3_, CF_3_ and C_5_H_5_ groups [35]. The nevertheless constrained environments within a solid structure open up the possibilities of added reaction selectivity, potentially different from that observed in solution. For example, if a reaction cavity could be designed to be chiral then asymmetric reactivity may also be possible [36]. As solid-state reactions proceed, the products will replace the reagents. This generally occurs from the surface downwards, and Kaupp [37] has proposed the idea of ‘phase rebuilding’ where reaction direction is determined by crystallographic faces, and lattice reconstruction occurs over thousands of angstroms at a time. The kinetics of solid-state reactions have proved difficult to measure owing to their inherent complexity, as the reaction may take place at different rates upon the surface and within the interior of a crystalline material. For example, Caulton and co-workers [38] proposed that changes in molecular shape on reaction induce strain in a crystalline material, which in turn promotes micro-cracking. Such cracks will expose more of the interior of the crystal to the gaseous reagent and increase reaction rate. When little change in shape occurs the crystals may become ‘passivated’ by a surface layer of the product, slowing further reactivity.

## 4. Stoichiometric solid–gas reactions

A very early example of solid-state organometallic synthesis using solid–gas techniques was reported in the 1960s when the oxidative addition of various HX gases (HF, HCl, HBr, HI and H_2_S) to Vaska-type complexes IrX′(CO)(PPh_3_)_2_ (X′=Cl, Br, I, SCN) was reported to form *trans*-Ir(PPh_3_)_2_(X′)(CO)(H)(X) [39]. Similarly, addition of I_2_ to Pt(acac)_2_ (acac=acetylacetonate) forms the oxidative addition product *trans*-PtI_2_(acac)_2_ [40]. More recently, Brammer and co-workers [41–43] have shown that the reaction of *trans*-[CuCl_2_(*n*-X-C_5_H_4_N)_2_] (*n*=3,4; X=Cl, Br) with HCl gas to form [*n*-X-C_5_H_4_NH]_2_[CuCl_4_] requires breaking of two Cu−N bonds to form Cu−Cl bonds and N−H bonds. These reactions have been monitored by powder diffraction, including *in situ* powder synchrotron diffraction [41], showing that microcrystalline products are formed.

**Scheme 1.**
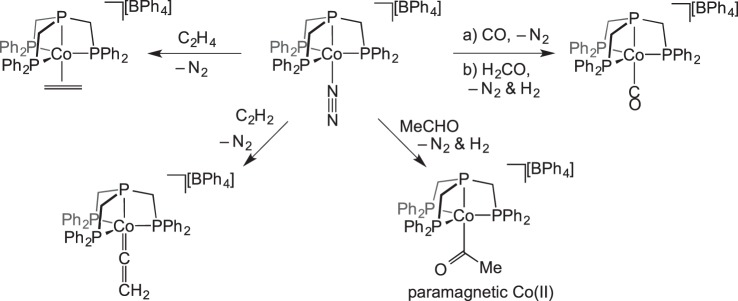
Solid-state gas exchange reactions facilitated by labile N_2_ ligands.

Organometallic stoichiometric reactions with gaseous reagents were extensively studied by Bianchini in the 1990s. For example, the displacement of labile N_2_ from 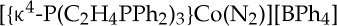 by various gases was studied in a solid–gas reaction (scheme 1) [44]. It was proposed that small gaseous reagents could penetrate the crystal lattice of [BPh_4_]^−^ anions by dissolving in the hydrophobic regions formed by the tetraphos ligand backbone and anion phenyl groups. As is shown later (§5), displacement of a labile N_2_ also occurs in single-crystal-to-single-crystal transformations.

**Scheme 2.**
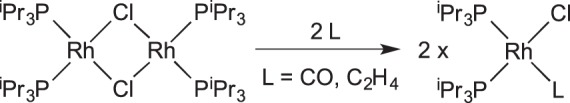
Solid–gas reactivity of a dimeric species to afford monomeric complexes.

**Scheme 3.**
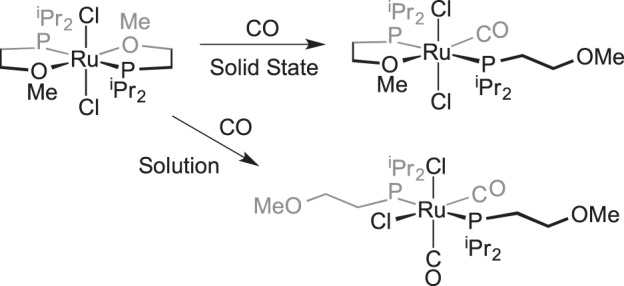
Hemilable ligand displacement in the solid state.

An interesting case of ligand displacement, in as much as that cleavage of a dimer is occurring in the solid state, comes from the reaction shown in scheme 2 in which Werner and co-workers [45] reported that addition of CO or ethene to a chloride-bridged Rh(I) dimer resulted in the generation of monomeric species, where two dative Rh–Cl bonds had been cleaved by CO or ethene. Other examples of ligand of displacement in the solid state have been reported. Werner and co-workers reported that addition of CO to a Rh-complex that contains a hemilabile [46,47] phosphine–ether ligand results in displacement of one Rh–ether linkage, whereas a dicarbonyl will form in the analogous solution-phase reaction (scheme 3) [45]. Addition of CO to Rh(PPh_3_)_3_(OAr) (e.g. Ar=C_6_Cl_5_) in the solid state results in an intermediate, tentatively described as five-coordinate Rh(PPh_3_)_3_(OAr)(CO), from which washing of the solid with ether removes PPh_3_ to afford *trans*-Rh(PPh_3_)_2_(OAr)(CO) [48]. Related five-coordinate species can be isolated from addition of CO in the solid state to square planar complexes such as [Ir(COD)(PPh_3_)(PhCN)][ClO_4_] to give, for example, [Ir(COD)(PPh_3_)(CO)_2_][ClO_4_] via loss of PhCN [49]. Milstein and co-workers [50] have shown that CO can bind reversibly to a 16-electron Rh(I) nitrosyl pincer complex, in which an equilibrium is established between a five-coordinate, CO-bound, and four-coordinate, CO-free, complex. Concomitant with this addition of CO is the change in NO binding mode from linear to bent, as measured by infrared (IR) spectroscopy.

**Scheme 4.**
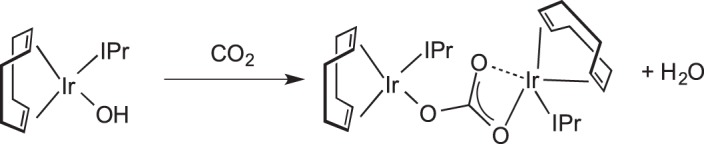
CO_2_ fixation using an Ir-hydroxide.

The reversible addition of CO_2_ to the rhodium and iridium complexes M(CO)(PPh_3_)_2_(OH) was reported by Flynn & Vaska [51], although the nature of the M⋯CO_2_ interaction was not clarified. Very recently, Nolan and co-workers [52] have reported that CO_2_ rapidly (2 min) inserts into the O−H bond of Ir(COD)(IPr)OH (IPr=1,3-di-isopropyl-imidazolin-2-ylidene) in a solid–gas reaction to form a bimetallic carbonate, 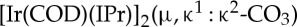, scheme 4, with the concomitant elimination of water [IPr=1,3-bis(isopropyl)imidazol-2-ylidene)].

**Scheme 5.**
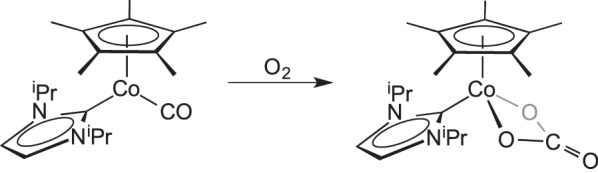
Aerobic oxidation to a carbonyl to form carbonate complex.

A related carbonate complex that comes from the aerobic oxidation of (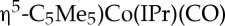 to form a carbonato complex 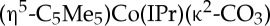 was reported by Radius and co-workers [53]. This process occurs rapidly in air, both in solution and in the solid phase (scheme 5).

**Scheme 6.**
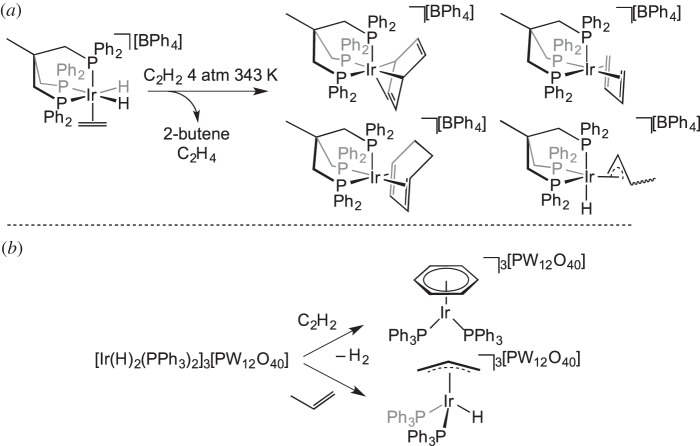
(*a*,*b*) Reaction of cationic iridium complexes with alkenes and alkynes in the solid state resulting in C–C couplings and/or C–H activation.

Addition of alkynes and alkenes to a metal complex may result in olefin oligomerization through C−H and C−C coupling processes, common transformations in solution-phase organometallic chemistry [1]. Bianchini *et al.* [54,55] demonstrated that addition of C_2_H_2_ to [Ir(triphos)(C_2_H_4_)(H)_2_][BPh_4_] [triphos=(Ph_2_PCH_2_)_3_CCH_3_] in the solid state leads to various products, including benzene and butadiene complexes (scheme 6*a*). Siedle & Newmark [56] have demonstrated trimerization of C_2_H_2_ with [Ir(H)_2_(PPh_3_)_2_]_3_[PW_12_O_40_] to form a benzene complex, whereas C−H activation of propene with the same organometallic starting material forms an allyl complex (scheme 6*b*). Related transformations in which bound ethene in a Rh(I) tripyrazolylhydroborate complex undergoes C−H and C−C bond-forming processes to form allylic species have been reported by Carmona and co-workers [57]. C−Br activation in an η^2^-azobenzene ligand coordinated to a {Pt(PEt_3_)_2_} fragment occurs in the solid state to give the corresponding Pt-aryl-halide [58]. The oxidative addition of a C−Cl bond is reported in the solid-state transformation of the zwitterionic Rh(I) η^6^-arene complex 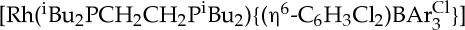 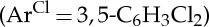 into the dimeric Rh(III) complex 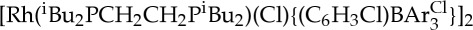 [59]. Interestingly, this process generates two isomers in the solid state, whereas the same process in solution only accesses the thermodynamic isomer. Bond isomerizations in the solid state, involving C−C cleavage, between Ru-alkynylketones and Ru-vinylidenes have been followed using IR spectroscopy, and kinetic data were obtained for this transformation. This is a very rare example of such an analysis of reactivity in solid-state organometallic chemistry, and, from these data, the authors propose a mechanism controlling the reaction that invokes nucleation and nuclei growth rather than diffusion, chemical reaction or phase boundary-controlled steps [60].

**Scheme 7.**
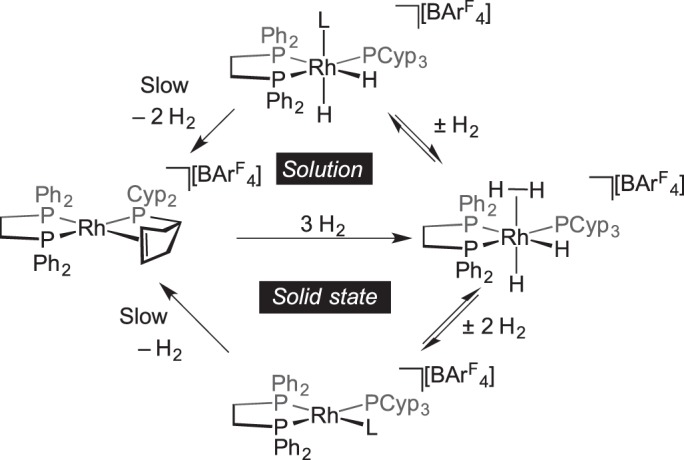
Reversible addition of hydrogen and C–H activation in solution and the solid state. L=agostic or solvent interactions.

Reversible hydrogen addition to metal complexes is of considerable interest with regard to processes such as hydrogenation, hydrodesulfurization and hydrogen storage applications [1]. The reversible addition of three molecules of hydrogen to 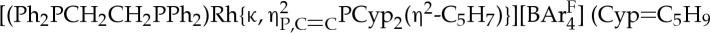, 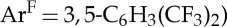 to form [(Ph_2_PCH_2_CH_2_PPh_2_)Rh(PCyp_3_)(H)_2_(H_2_)][BAr^F^_4_] has been reported, in which a dihydrogen ligand is coordinated, and the phosphine–alkene ligand has been hydrogenated [61]. In the solid phase, the product loses two molecules of hydrogen under application of a vacuum to form a red intermediate proposed to be the Rh(I) complex 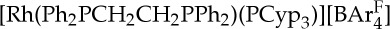, in which an agostic interaction from the phosphine is proposed. This red intermediate then slowly loses a further equivalent of H_2_ via an alkyl dehydrogenation (C−H activation and β-elimination) to reform 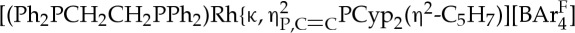. In solution, no red Rh(I) intermediate is observed, and removal of the dihydrogen ligand under vacuum results, instead, in a Rh(III) dihydride (scheme 7).

**Scheme 8.**
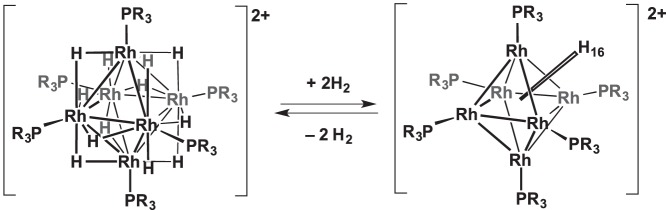
Reversible H_2_ addition to 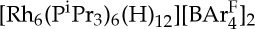. R=^i^Pr.

The simple reversible addition of dihydrogen to monometallic [62] and multi-metallic cluster [63–66] species in the solid state has been reported by a number of groups. In these instances, the H_2_-free complexes are often electronically unsaturated and stabilized by bulky ligands around the metal centre(s). An example shown in scheme 8 is the reversible addition of two molecules of H_2_ to the cluster 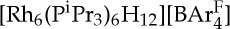 to give 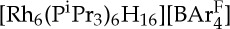 [67]. Related coordinate unsaturation in clusters enabled via loss of a weakly bound ligand in the solid state (such as NCMe) allows for solid–gas reactivity of Os_3_(CO)_11_L (L=NCMe) with CO, NH_3_ and H_2_ [68].

Deliberate installation of coordinate and electronic unsaturation at a metal centre means that solid–gas reactions can be made particularly facile. Caulton and co-workers have reported that 16-electron Ru(CO)_2_(P^t^Bu_2_Me)_2_ adds H_2_, Cl_2_ or O_2_ to give the corresponding 18-electron complexes *cis*,*cis*,*trans*-Ru(H)_2_(CO)_2_(P^t^Bu_2_Me)_2_, *cis*,*cis*,*trans*-Ru(Cl)_2_(CO)_2_(P^t^Bu_2_Me)_2_ and Ru(η^2^-O_2_)(CO)_2_(P^t^Bu_2_Me)_2_, respectively. Interestingly, reaction with HSiMe_3_ gave the dihydride Ru(H)_2_(CO)_2_(P^t^Bu_2_Me)_2_ dissolved in liquid Me_3_SiSiMe_3_, formed by a dehydrocoupling process. Reaction with CO is particularly slow, and this is postulated to be due to the fact that the product, Ru(CO)_3_(P^t^Bu_2_Me)_2_, is a similar size to the organometallic reactant, and this results in surface passivation rather than the fracturing of the crystal (and concomitant faster ingress of gas) that is suggested to occur when product and starting material geometries are different [38]. The electronically and coordinatively unsaturated complex 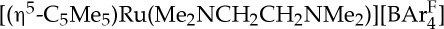 adds H_2_, N_2_, O_2_, CO and ethylene in the solid state, with the latter shown to be reversible. These new complexes (except the CO adduct) were unstable in solution and were characterized by elemental analysis [69].

## 5. Single-crystal-to-single-crystal transformations

A single-crystal-to-single-crystal (SC−SC) transition is one in which crystallinity is retained throughout a reaction, allowing the product to be characterized directly by single-crystal X-ray crystallography without recourse to recrystallization from solution [32]. For crystallinity to be retained, only a very small structural reorganization can be tolerated to avoid the break-up of the lattice. Crystal size also probably affects reaction time in SC−SC transitions because of surface area to volume ratio implications. Molecular designs that enable such transitions to take place involve use of bulky ligands or anions which dominate the packing and thus can create a rigid, porous, structure in which smaller movements around the metal centre are made possible [13,70]. SC−SC reactions present excellent possibilities for selectivity to be controlled within a solid-state environment, as the reaction cavity necessarily must remain well defined throughout to preserve crystallinity.

**Scheme 9.**
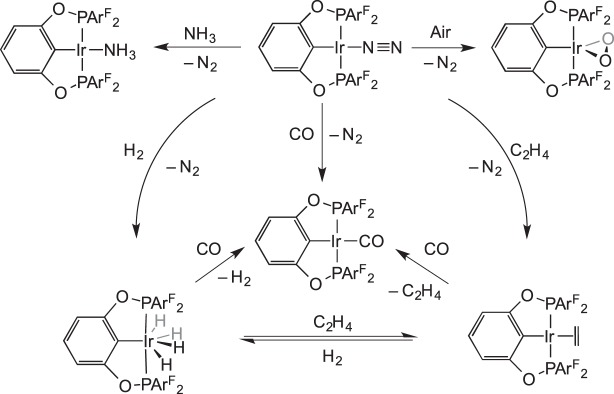
SC–SC transitions using Ir–pincer complexes. Ar^F^=2,4,6-C_6_H_2_(CF_3_)_3_.

If gaseous reagents require access to the interior of the crystal then empty or partially filled channels throughout the lattice may be necessary [33]. This was suggested by Brookhart and co-workers [13], who noted the channels of disordered toluene throughout the crystalline lattice of (POCOP)IrL (POCOP=1,3-[OP{2,4,6-C_6_H_2_(CF_3_)_3_}_2_]_2_C_6_H_3_, L=N_2_, CO, NH_3_, C_2_H_4_, O_2_). They reported a series of SC−SC gas transfer reactions using this system (scheme 9). Interestingly, the precursor complex studied (L=N_2_) is stable under vacuum, but readily reacts with more strongly binding gases, which suggests that an associative mechanism for gas exchange is operating within the interior of the crystal. These SC−SC transformations presumably occur as the packing in the lattice is dominated by the large tris-(CF_3_) substituted aryl groups on the pincer ligand, meaning that changes in the ancillary ligands around Ir (e.g. CO for N_2_) result in minimal structural reorganization.

**Scheme 10.**
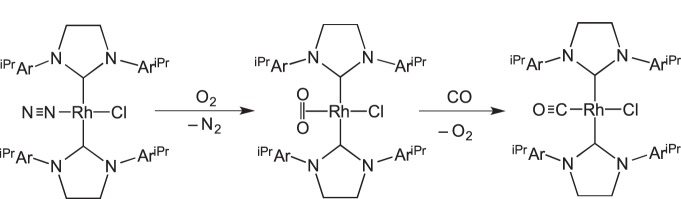
Sequential SC–SC gas transfer transitions. Ar^iPr^=2,6-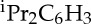.

Such transitions can also be reversible. van Koten and co-workers [71,72] reported the reversible addition of SO_2_ to (NCN)PtCl (NCN=C_6_H_2_-5-(OH)-1,3-(CH_2_NMe_2_)_2_), which induced significant changes in the geometry of the metal centre, from square planar to pseudo-square pyramidal, and remarkably such a large structural change does not result in the loss of crystallinity, albeit these processes occur in the microcrystalline powder state and the transformations are monitored by powder diffraction techniques and IR spectroscopy rather than by single-crystal X-ray diffraction (crystals suitable for such analysis were grown independently). The authors speculate that these processes are likely to involve local solutions of the reactants that recrystallize at a comparable rate to solute formation. They proposed that this material could be used as a gas-triggered switch, with SO_2_ uptake signalled either by colour change or by crystal expansion. SC−SC transitions may also occur sequentially. In an elegant example, Crudden and co-workers [70] presented a double SC−SC gas exchange reaction using the (S^*I*^Pr)_2_RhCl(L) system 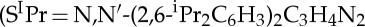, L=N_2_, O_2_ or CO; scheme 10). First, N_2_ is replaced with O_2_ to give a dioxygen adduct, and then O_2_ is replaced with CO, without crystal degradation at either stage.

Alcohol uptake (i.e. MeOH, EtOH and iPrOH) by the non-porous coordination polymer [Ag_4_(O_2_C(CF_2_)_2_CF_3_)_4_(TMP)_3_]_*n*_ (**A**) is reversible in a SC−SC transformation by a solid–vapour process (TMP=2,3,5,6-tetramethylpyrazine). In an elegant sequence of substitution reactions, consecutive alcohols are introduced into the lattice: A-EtOH 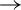 A-MeOH 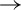 A-^i^PrOH 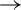 A-EtOH without loss of single crystallinity. These ligand substitution reactions are accommodated by changes in coordination geometry at specific Ag(I) centres, and specifically alcohol insertion occurs into one-quarter of the Ag−O carboxylate bonds [73,74]. As this material does not have significant porosity, a mechanism is proposed in which concerted motion of the disordered fluoroalkyl chains allows for the transport of the alcohol molecules within the crystals. Powder X-ray crystallography techniques were also used to follow these transformations.

**Scheme 11.**
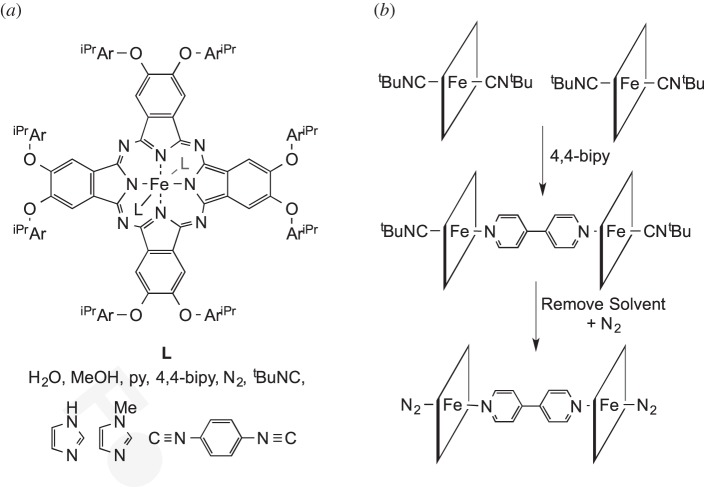
(*a*) Porous phthalocyanine–derivative complex (PNC[*v*L–Fe–*c*L]) which can undergo SC–SC ligand (L) exchanges when immersed in organic solvents. Ar^iPr^=2,6-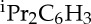. (*b*) Example of two sequential ligand exchanges, displaying the linking of two metal centres by a bidentate ligand (phthalocyanine derivative simplified to a flat square).

SC−SC transitions can also take place in a suspension of non-solvating liquid, for example in polymeric platform materials. McKeown and co-workers [33] reported such SC−SC transitions using Fe(MeOH)_2_(phthalocyanine). Large interconnected voids (8 nm^3^) run through these structures that are defined by a cubic assembly of six of the phthalocyanine groups. These voids allow liquid penetration, and axial ligands can be reversibly, and rapidly, displaced by a variety of exogeneous ligands (scheme 11). Interestingly, monodentate ligands bind preferentially to an axial binding site within this cubic assembly, whereas bidentate ligands selectively bind to link neighbouring cubic assemblies together. Selective exchange between water and methanol has been observed as a SC−SC transformation in the trinuclear iron complexes [Fe_3_(μ_3_-O)(μ_2_-CH_3_COO)_6_(C_5_H_5_NO)_2_(L)][ClO_4_] (L=H_2_O, MeOH) [75], whereas heterolytic dissociation of water at a bis(μ-oxo)divanadiumpolyoxometallate, which models the interactions of water with metal oxide surfaces, was found to occur as a SC−SC transformation.

**Scheme 12.**
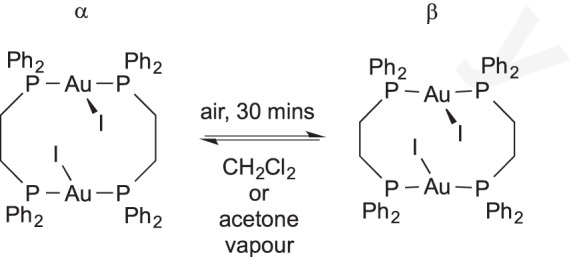
Phase change SC–SC transition driven by drying in air and reversible by exposure to CH_2_Cl_2_ or acetone vapour.

In the absence of added reagents, SC−SC transitions can occur in the form of a simple phase change within the crystal lattice. Balch and co-workers [77] reported a reversible phase change when acetone or dichloromethane vapour is passed over a crystal of β-Au_2_(μ-Ph_2_PCH_2_CH_2_PPh_2_)_2_I_2_⋅(OCMe)_2_, even though no additional solvent incorporation is observed. Two equivalents of acetone are present in both phases. The reverse reaction occurs if the crystal is left in air (scheme 12).

**Scheme 13.**
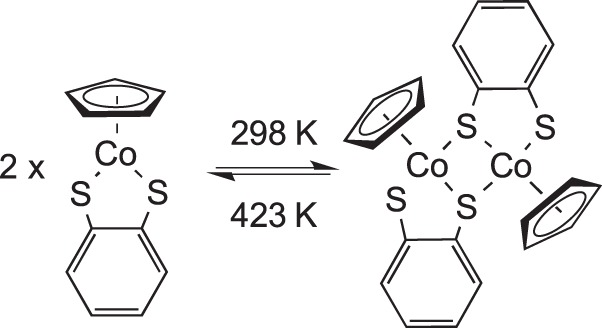
Reversible dimerization in a single crystal.

Brill, Rheingold and co-workers [78] of 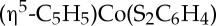 in the single-crystal phase at room temperature could be reversed upon warming to 423 K while still maintaining crystallinity (scheme 13). The dimeric species is thermodynamically favoured in the crystalline lattice at room temperature, but the monomer is favoured thermodynamically in solution, presumably owing to a dominant entropy term in solution.

**Scheme 14.**
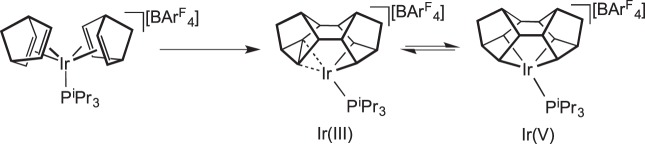
Formation of a BINOR-S complex and reversible SC–SC C–C cleavage.

Another SC−SC transformation that occurs without additional reagent is the reversible C−C activation in 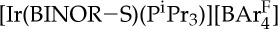, which contains a rare example of an agostic M ⋯ C −C bond (BINOR-S=1,2,4,5,6,8-dimetheno-s-indacene). In this complex, reversible C−C cleavage occurs to form an equilibrium mixture, in the crystalline phase, of dynamically disordered C−C activated [Ir(V)] and C−C agostic [Ir(III)] complexes—the ratio of which changes with temperature. Interestingly, the 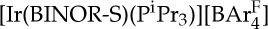 complex is itself made from a solid-state organometallic reaction, where cyclodimerization of two norbornadiene ligands occurs to yield the BINOR-S ligand (scheme 14) [79].

**Scheme 15.**
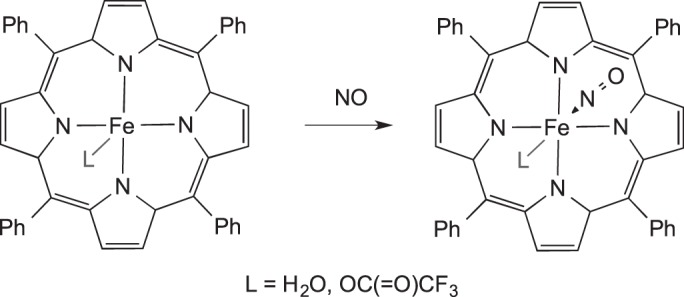
Formation (NO)–Fe–porphyrin complexes by SC–SC transitions.

SC−SC reactions present possibilities for directly forming reactive complexes, which may not be accessible cleanly using solution routes. C−N oxidative cleavage in the PNP pincer complex 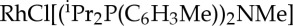 to give 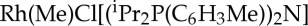 occurs in the solid state in a SC−SC transformation [80]. Saliently, when performed in the bulk, this preparative route is cleaner than that in solution [81]. Richter-Addo and co-workers [82] were able to crystallographically characterize biologically important porphyrin complexes which bind NO. Examples include (TPP)Fe(NO)(OC(=O)CF_3_), [(TPP)Fe(NO)(H_2_O)][(TPP)Fe(H_2_O)][OC(=O)CF_3_]_2_ [83] (TPP = tetraphenylporphyrin) and [(oep)Fe(NO)(S-2,6-(CF_3_CONH)_2_C_6_H_3_)] [84] (oep = octaethylporphyrinato dianion). These complexes were formed by addition of NO gas to the unsaturated precursors in the solid state. This transformation was not possible using solution crystallization methods, as in solution a mixture of by-products form instead (scheme 15) [82–84].

**Scheme 16.**
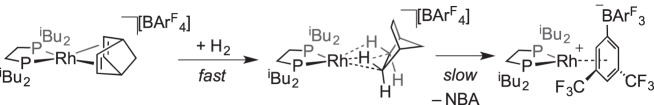
Formation of an alkane complex in the solid state.

Perhaps the most dramatic exploitation of solid-state SC−SC transformations in stabilizing highly reactive complexes comes from the addition of H_2_ to the complex [Rh(NBD)(^i^Bu_2_PCH_2_CH_2_P^i^Bu_2_)][BAr^F^_4_] (NBD=C_7_H_8_, Ar^F^=3,5-C_6_H_3_(CF_3_)_2_) that results in the isolation and structural characterization of a transition-metal sigma–alkane complex in which the hydrogenated organic fragment (NBA=C_7_H_12_) binds to the metal through two Rh⋯ H−C sigma interactions [Rh(η^2^,η^2^-NBA)(^*i*^Bu
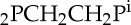
Bu_2_)][BAr^F^_4_] (scheme 16) [85]. Such alkane complexes are exceedingly rare, with only two examples having been previously characterized in the solid state by single-crystal X-ray diffraction, in which there is a close approach of the alkane H−C bond to a metal centre [86,87]. Ultimately, this alkane complex undergoes a further transformation, in the solid state, to form a complex in which the alkane ligand has been lost and the [BAr^F^_4_]^−^ anion coordinates. An intermediate, mono-hydrogenated, species was proposed 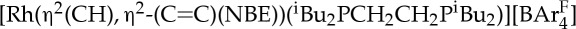 (NBE=C_7_H_10_), which also adds H_2_ in the solid state to give the alkane complex, as shown by solid state NMR spectroscopy.

### (a) Overview of the structural changes associated with single-crystal-to-single-crystal transformations

Selected examples of SC−SC transitions are displayed in table 1 with their respective crystallographic volume change (*z*=1 equivalent). Minimal structural changes are apparently necessary to allow for SC−SC transitions, and most examples reported show less than 4% volume change in the lattice volume. The outlier of van Koten and co-workers’ example of SO_2_ uptake is exceptional because it involves a very large percentage volume change (15.7%), with expansion predominantly along one axis. However, this occurs in the microcrystalline phase without loss of crystallinity as measured by powder diffraction, and SC−SC experiments were not successful. Noteworthy is the 10% change in volume associated with reversible alcohol addition in the Ag-carboxylates, this perhaps being associated with the fact that this material is a coordination polymer in the solid state, which might allow for increased flexibility without loss of crystallinity.

**Table 1.** Examples of SC–SC transitions and the crystallographic volume changes involved. POCOP=1,3-[OP{C_6_H_2_(CF_3_)_3_-2,4,6}_2_]_2_C_6_H_3_; NCN=C_6_H_2_-5-(OH)-1,3(CH_2_NMe_2_)_2_; S^i^Pr=N,N′-(2,6-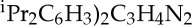; TPP= tetraphenylporphyrin; oep=octaethylporphyrinato dianion; NBD=C_7_H_8_; NBA= C_7_H_12_; Ar^F^=3,5-C_6_H_3_(CF_3_)_2_; TMP=2,3,5,6-tetramethylpyrazine.

	entry	starting complex space group, volume (Å^3^), *z*	product space group, volume (Å^3^), (*z*)	[change in volume (Å^3^)] *and percentage change*. For *z*=1 equivalent
**1**	Brookhart and co-workers [13]	(POCOP)Ir(N_2_); 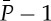 , 3002.11(14), *z*=2	(POCOP)Ir(O_2_); 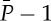 , 3046.3(10), *z*=2	[+22] *+1.4%*
			(POCOP)Ir(CO); 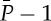 , 3000.1(2), *z*=2	[−1] *−0.06%*
			(POCOP)Ir(C_2_H_4_); 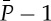 , 3020.6(2), *z*=2	[+9] *+0.6%*
			(POCOP)Ir(H)_2_(H_2_); 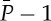 , 2986.6(4), *z*=2	[−8] *−0.6%*
			(POCOP)Ir(NH_3_); 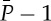 , 3003.56(19), *z*=2	[+1] *+0.04%*
**2**	van Koten and co-workers [71]	(NCN)PtCl; *P*na2_1_, 1346.0(4), *z*=4	(NCN)PtCl(SO_2_); *P*na2_1_, 1557.5(4), *z*=4	[+53] *+15.7%*
**3**	Crudden and co-workers [70]	(S^I^Pr)RhCl(N_2_); *P*2_1_2_1_2, 2855.15(8), *z*=2	(S^I^Pr)RhCl(O_2_); *P*2_1_2_1_2, 2852.13(12), *z*=2	[−3] *−0.1%*
		(S^I^Pr)RhCl(O_2_); *P*2_1_2_1_2, 2852.13(12), *z*=2	(S^I^Pr)RhCl(CO); *P*2_1_2_1_2, 2861.5(3), *z*=2	[+9] *+0.4%*
**4**	McKeown and co-workers [33]	PNC[*c*^*t*^BuNC-Fe-*v*^*t*^BuNC]; *P*n–3n, 52348.5(7), *z*=12	PNC[*c*^*t*^BuNC-Fe-*v*(py)_0.7_(^*t*^BuNC)_0.3_]; *P*n–3n, 52736(2), *z*=12	[+32] *+0.7%*
			PNC[*c*MeOH-Fe-*v*Me(C_3_N_2_H_3_)]; *P*n–3n, 52819.6(12), *z*=12	[+39] *+0.8%*
			PNC[*v*^*t*^BuNC-Fe-*c*(4,4-bipy)-Fe-*v*^*t*^BuNC)]; *P*n–3n, 54263.4(5), *z*=12	[+160] *+3.7%*
			PNC[*v*N_2_-Fe-*c*CN(C_6_H_4_)NC-Fe-*v*N_2_)]; *P*n–3n, 53204(5), *z*=12	[+71] *+1.6%*
**5**	Balch and co-workers [77]	α-Au_2_(μtPh_2_PCH_2_CH_2_-PPh_2_)_2_I_2_.(OCMe)_2_; *P*2_1_/c, 2824.28(13) *z*=2	βAu_2_(μ–Ph_2_PCH_2_CH_2_PPh_2_)_2_I_2_.(OCMe)_2_; 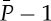 , 1411.99(7), *z*=1	[−0.2] *∼0%*
**6**	Brill, Rheingold and co-workers [78]	(η^5^-C_5_H_5_)Co(S_2_C_6_H_4_); *P*2_1_/c, 2159.1(6), *z*=8	[(η^5^-C_5_H_5_)Co(S_2_C_6_H_4_)]_2_;*P*2_1_/c, 1031.2(3), *z*=*2* (*dimer*)	[−12] *−4.5%*^a^
**7**	Richter-Addo and co-workers [82–84]	(TPP)Fe{OC(=O)CF_3_}; 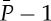 , 2093.1(3), *z*=2	(TPP)Fe(NO){OC(=O)CF_3_}; 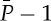 , 2144(3), *z*=2	[+25] *+2.4%*
		[(TPP)Fe(H_2_O)][OC(=O)CF_3_]; 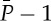 , 1833.4(3), *z*=2	[(TPP)Fe(NO)(H_2_O)][(TPP)Fe(H_2_O)] [OC(=O) 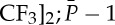 , 3818.2(16), *z*=2	[+38] *+4.1%*^b^
		[(oep)Fe{S-2,6-(CF_3_CONH)_2_C_6_H_3_}]; 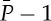 , 2266.3(6), *z*=2	[(oep)Fe(NO){S-2,6-(CF_3_CONH)_2_C_6_H_3_}]; 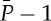 , 2360(2), *z*=2	[+47] *+4.1%*
**8**	Weller and co-workers [85]	[Rh(^i^Bu_2_P 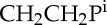 Bu_2_)(η^2^η^2^-NBD)][BAr^F^_4_]; *C* 2/c, 5957.62(18), *z*=4	[Rh(^i^Bu_2_P 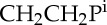 Bu_2_)(η^2^η^2^-NBA)][BAr^F^_4_]; *C* 2/c, 6044.1(3), *z*=4	[+22] *+1.5%*
**9**	Ozerov and co-workers [80,81]	RhCl[(^i^Pr_2_P(C_6_H_3_Me))_2_NMe]; *P*2_1_2_1_2_1_, 5955(4), *z*=8	Rh(Me)Cl[(^i^Pr_2_P(C_6_H_3_Me))_2_N]; *P*2_1_2_1_2_1_, 5976(4), *z*=8	[+21] *+0.4%*
**10**	Brammer and co-workers [74]	[Ag_4_(O_2_C(CF_2_)_2_CF_3_)_4_(TMP)_3_]_*n*_; 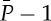 , 1405.34(6), *z*=1	[Ag_4_(O_2_C(CF_2_)_2_CF_3_)_4_(TMP)_3_(^i^PrOH)]_*n*_; 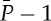 , 1545.9(2), *z*=1	[+140.6] *+10.0%*

## 6. Catalysis in the solid state

### (a) Heterogeneous organometallic catalysts

The heterogenization of single-site catalysts brings together the benefits of heterogeneous catalysis (i.e. recyclability and ease of removal from the reaction mixture) with the potential for intimate control over transformations that occur at the metal centre that is provided by the local ligand environment in a homogeneous system [15,16]. There are a number of strategies that can be used to facilitate the heterogenization of well-defined organometallic catalyst systems, including surface-supported organometallic chemistry in which a platform material such as silica, zeolites or metal oxides support directly, or indirectly via linker groups, the organometallic complex [88–98]. Metal organic frameworks (MOFs) are also particularly attractive as one-, two- and three-dimensional assemblies can be created in which the metal atoms often act as the geometry-enforcing linkage points, but can also be envisaged as potential active sites for catalysis [24]. In addition, a MOF may simply act as a reaction cavity, with the organometallic species as a host/guest material [99]. Within the context of this review that concentrates on the reactivity of well-defined organometallic complexes in the solid state these materials, as they are often not well defined at the metal centre of interest, are not included here.

### (b) Self-supported organometallic catalysis

**Scheme 17.**
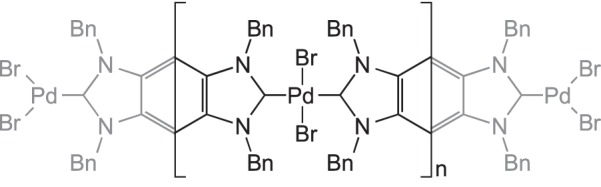
A coordination polymer with Pd capable of catalysing heterogeneous Suzuki–Miyaura cross coupling in water.

Self-supported catalysts invoke an active metal centre in which the ligand environment also acts as the platform microporous material [100]. The advantage in many of these systems, compared with heterogenized organometallic catalysts, is that they are well defined at the metal–centre and thus more amenable to structural and spectroscopic investigation. Early reports of self-supported catalysts include the linkage polymers of [RhCl(CO)(1,4-(CN)_2_C_6_H_4_)]_*n*_ which can hydrogenate and isomerize 1-hexene, with no leaching of complexes into solution [101]. The active rhodium site was created by photolytic dissociation of the CO ligand. A similar system was formed with two bridging ligands per metal, enabling a well-defined three-dimensional-stacked layer structure to form. The surface and corner positions of this structure are likely to contain unsaturated metal centres which are catalytically active; however, the interior sites are proposed to be totally inactive [102,103]. Multi-dentate oxime, thiourea, phosphine and NHC ligands have been used to form frameworks with Pd-centres for use in Suzuki–Miyaura C−C coupling reactions [104–107]. For example, Karimi & Akhavan [107] reported a coordination polymer of palladium with a linking bidentate NHC ligand, which is insoluble in water, resulting in C−C coupling catalysis that could be performed using water as the substrate and product solvent (scheme 17). Although the authors used the mercury test, which probes for nanoparticle formation, which showed no loss in activity, it is difficult to unequivocally prove that nanoparticles are in no way involved for such systems.

**Scheme 18.**
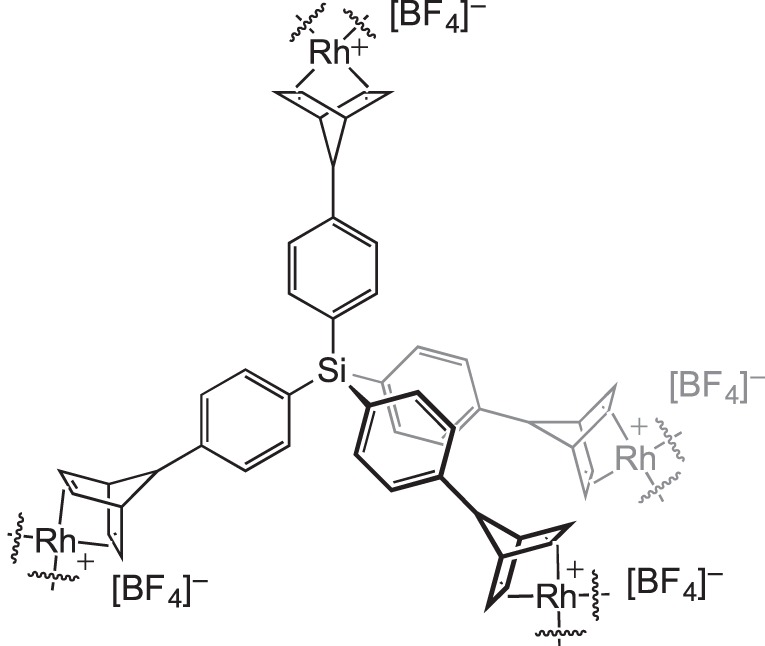
A microporous organometallic framework based upon rhodium alkene coordination.

More complex microporous structures can also be formed with a mixture of metal sites. For instance, copper, nickel or palladium porphyrin moieties can be combined with rhodium–polycarboxylate linkages in which both metal sites may exhibit cooperative effects for hydrogenation reactions [108,109]. Kaskel and co-workers [110] have recently reported the formation of a microporous organometallic network based upon a rhodium alkene fragment linked to a rigid tetraphenylsilane backbone (scheme 18). While an accurate structural determination has proved difficult, the framework appeared to be air-stable unlike its homogeneous analogue [Rh(NBD)_2_][BF_4_]. This material catalysed transfer hydrogenation reactions.

### (c) Solid-state organometallic catalysis without a support

**Scheme 19.**
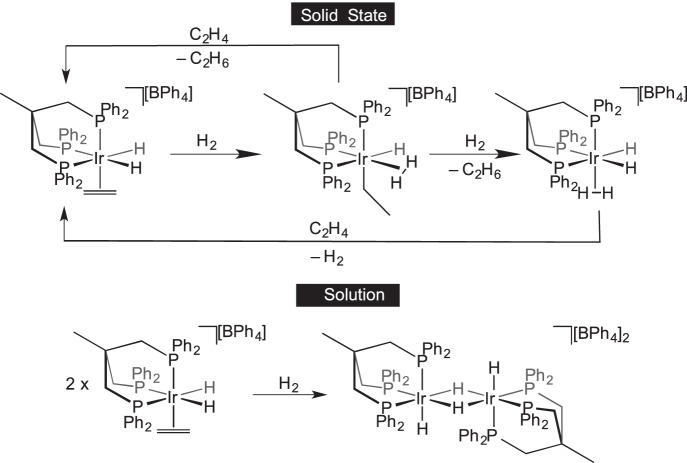
Catalytic ethene hydrogenation in the solid state versus solution.

**Scheme 20.**
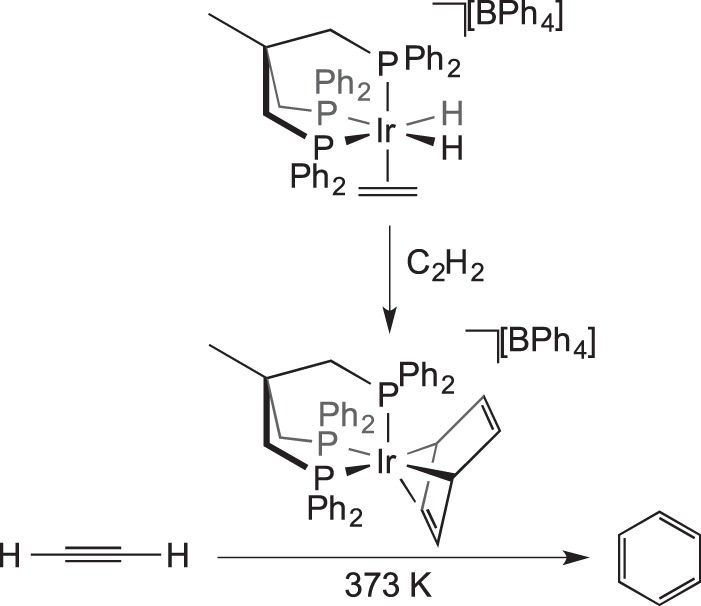
Trimerization of ethyne using a solid-state catalyst.

Solid-state catalysis using well-defined organometallic complexes that are not incorporated into a platform material is a relatively undeveloped field. Bianchini *et al.* [14] introduced the concept with simple ethene hydrogenation reactions using [(triphos)Ir(H)_2_(C_2_H_4_)][BPh_4_] at 343 K (scheme 19). The catalyst was active in the solid state, in a mechanism proposed to operate via hydride migration to form an Ir−(C_2_H_5_) species, which can react with further H_2_ followed by reductive elimination of ethane. In solution, the same species was not catalytically active, because a coordinatively saturated dimeric bridging hydride species rapidly forms in the presence of H_2_ which was inactive for further reactions. Although some of the inactive dimeric species is also formed in the solid-state reaction, it appears to form at a slower rate than in solution. This highlights the ability of the solid state to maintain the integrity of the reactive species by playing a role in protecting them from deactivation pathways that require structural reorganization. The [BPh_4_]^−^ anions are proposed to create a hydrophobic lattice structure ideal for allowing the passage of small hydrocarbon gases. The catalytic trimerization of ethyne to form benzene was also investigated by Bianchini and co-workers, who showed that a η^4^-benzene complex (formed itself from a solid–gas reaction) is an active pre-catalyst active at 373 K in the solid state (scheme 20) [55].

**Scheme 21.**
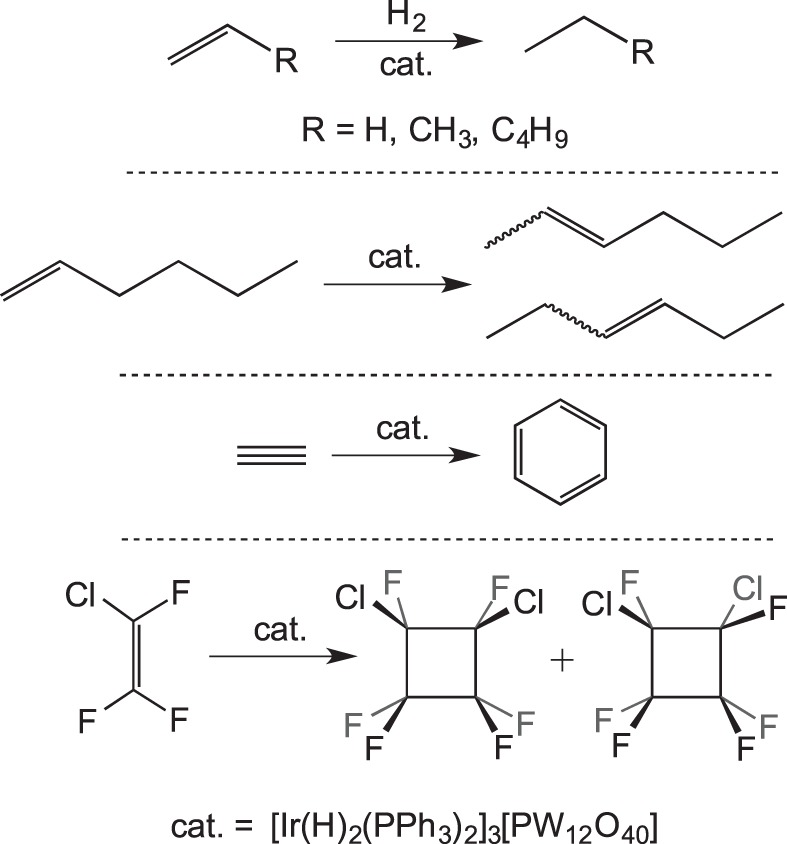
Catalytic reactions using [Ir(H)_2_(PPh_3_)_2_]_3_[PW_12_O_40_].

Siedle & Newmark [56] reported the room temperature catalytic activity of iridium phosphine cations partnered with Keggin-type trianions, [Ir(H)_2_(PPh_3_)_2_]_3_[PW_12_O_40_] (scheme 21), with the hydrogenation of ethene, propene and 1-hexene demonstrated. The isomerization of 1-hexene to a mixture of *cis-* and *trans*-2-hexenes and 3-hexenes was also reported, presumably via reversible C−H activation accessing an allyl-iridium–hydride intermediate. The authors do not comment on the rates of reaction. Similar to the findings of Bianchini, addition of excess ethyne forms benzene in catalytic quantities, with an iridium–benzene complex expected to act as the pre-catalyst, although the reaction is reported to be slow. The catalytic dimerization of CF_2_=CFCl to *cis-* and *trans*-1,2-dichlorohexafluorocyclobutane complexes was also reported.

**Scheme 22.**
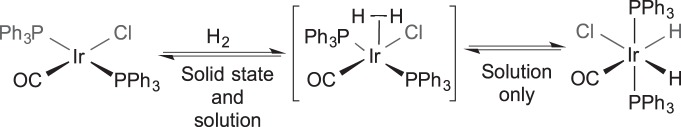
Reaction of Vaska’s complex with H_2_ in the solid state and solution.

Limbach and co-workers [111] have reported on the solid-state catalysed hydrogenation of ethene using Vaska’s complex, Ir(CO)Cl(PPh_3_)_2_, by following the reaction products by gas-phase ^1^H NMR spectroscopy. In solution, the product of H_2_ addition (which presumably related to the active catalyst) is a *cis*-dihydride/*trans*-phosphine species. In the solid state, this is not formed, and it was proposed that this was due to ligand reorientation being inhibited. The authors thus suggested a different pathway for hydrogenation in the solid state and solution (scheme 22) that invokes a dihydrogen intermediate as the active species in the solid state.

**Scheme 23.**
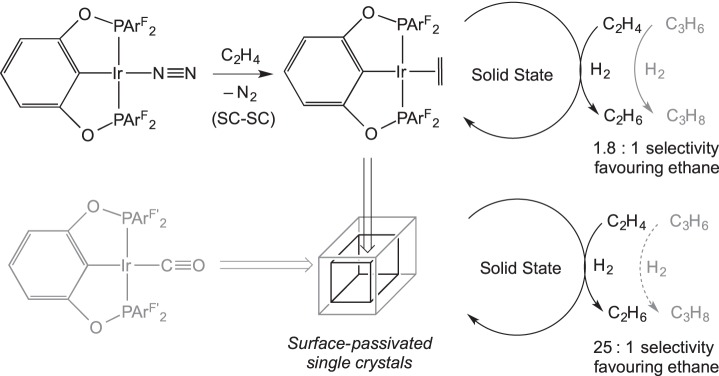
Hydrogenation of ethene using single crystals, and the selective hydrogenation of ethene in the presence of propene using surface-passivated single crystals.

Brookhart *et al.* [13] have reported the hydrogenation of ethene using single crystals of (POCOP)Ir(N_2_), as monitored by gas-phase NMR spectroscopy. At 298 K, the reaction requires 5 h to reach 95% conversion, but at 348 K 99% conversion occurs within 30 min. At this higher temperature, lattice-incorporated toluene is lost, and this is proposed to be responsible for the higher activity. (POCOP)Ir(C_2_H_4_) is the suggested resting state. Remarkable selective catalytic hydrogenation inside single crystals was also presented. By passivating the surface sites of crystals of (POCOP)Ir(N_2_) with a layer of (POCOP)Ir(CO), an incomplete crystal-to-crystal transition occurs. The resulting material can selectively hydrogenate ethene in the presence of propene, with a 25:1 preference at 348 K (scheme 23) [13]. It was proposed that the porous crystals allow the smaller ethene and hydrogen molecules access to the active interior metal sites, whereas the larger propene molecules cannot penetrate the surface. In the absence of surface passivation, only a small selectivity is seen in favour of hydrogenation of ethene (1.8:1 ratio of ethane to propane produced at 298 K), consistent with this.

**Scheme 24.**
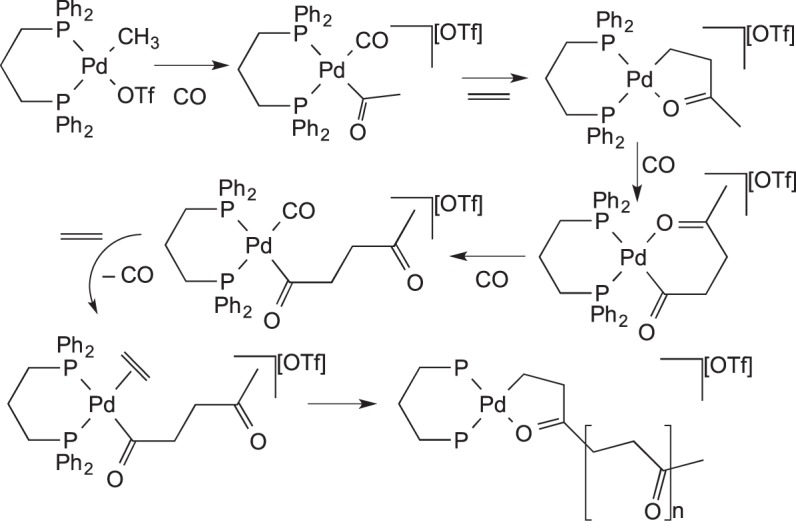
The proposed mechanism for the copolymerization of CO and ethene using a palladium catalyst in the solid state.

Mul and co-workers [112] have reported upon the palladium-catalysed CO/C_2_H_4_ co-polymerization reaction which is operated under gas phase or slurry conditions in industry. The catalyst becomes incorporated into the growing polymer chain, and so its nature as a heterogeneous or homogeneous catalyst is not well defined. Mul *et al.* chose to investigate the mechanism by using microcrystalline (Ph_2_PCH_2_CH_2_CH_2_PPh_2_)Pd(CH_3_)(OTf) deposited onto a gold surface. They were able to probe the first few turnovers of reaction using polarization modulation reflection–absorption IR spectroscopy (scheme 24). The findings suggest that ethene insertion into the Pd–acyl bond is actually CO-assisted. The incoming CO is able to displace the chelating ketone group more easily than ethene, but can itself then exchange with ethene. This subtlety had not been previously revealed by solution studies.

Dorta *et al*. [113] formed a variety of chiral crystalline organometallic complexes with either chiral phosphine ligands or chiral counterions, with the idea to investigate asymmetric catalysis. Unfortunately, this was not achieved, in this instance, and this interesting area has yet to be successfully developed.

## 7. Conclusion and future outlook

We hope that this review has shown that solid-state organometallic chemistry can offer significant advantages over the solution phase, especially when kinetic stability, or overall reaction selectivity, are different in the solid state compared with solution. Particularly exciting is the opportunity presented for SC−SC transformations in the solid state, as this methodology not only provides synthetic routes to new complexes but also enables direct structural analysis by single-crystal X-ray diffraction. However, for such transformations to proceed there is a requirement for minimal structural reorganization, and the appropriate design of systems that allow for this (e.g. large anions or bulky ligand groups) thus needs to be considered. An exciting prospect exists, which has not been developed to a significant extent, for such transformations to also result in catalytic processes. One example we suggest here is the selective transformation of hydrocarbons (alkane upgrading), where both solid–gas reactivity and selectivity from the local spatially well-defined environment will be important in producing effective and efficient catalysis. It will be fascinating to see how the field evolves as more examples of solid-state organometallic transformations are reported.
